# Child and Adolescent Psychosocial Support Programs Following Natural Disasters—a Scoping Review of Emerging Evidence

**DOI:** 10.1007/s11920-021-01293-1

**Published:** 2021-10-15

**Authors:** Gibbs L., Marinkovic K., Nursey J., Tong L. A., Tekin E., Ulubasoglu M., Callard N., Cowlishaw S., Cobham V. E.

**Affiliations:** 1 Melbourne School of Population and Global Health, University of Melbourne, Melbourne, VIC Australia; 2 Centre for Disaster Management and Public Safety, University of Melbourne, Melbourne, VIC Australia; 3 Phoenix Australia: Centre for Posttraumatic Mental Health, Melbourne, Australia; 4 Department of Economics, Deakin University, Burwood, VIC Australia; 5 School of Public Affairs, American University, Washington, D.C USA; 6 National Bureau of Economic Research (NBER), Cambridge, USA; 7 Institute of Labor Economics (IZA), Bonn, Germany; 8 Children’s Health Queensland Hospital and Health Service, Queensland, Australia; 9 School of Psychology, The University of Queensland, Queensland, Australia; 10 Children’s Health Queensland Child and Youth Mental Health Service, Queensland, Australia

**Keywords:** Child, Adolescent, Disaster, Mental health, Recovery, Intervention

## Abstract

**Purpose of Review:**

This review aimed to identify and describe evidence published in the past 3 years from trials of psychosocial support programs for children and adolescents affected by natural disasters.

**Recent Findings:**

Previous reviews have indicated these programs are beneficial overall. Positive impacts were documented in school-based programs conducted by trained teachers and paraprofessionals with stronger effects achieved by more qualified professionals.

**Summary:**

The review found supporting evidence for positive impacts of post-disaster psychosocial programs. However, the strength of evidence is limited due to heterogeneity in interventions and evaluations. The stepped care model was found to be useful in differentiating between programs and level of available evidence. Hobfoll’s five essential elements of mass trauma intervention provide an additional means of guiding program content and assessments, particularly for universal programs. Identified gaps in evidence included groups likely to be at most risk: preschool children, ethnically diverse groups, those with disability, and social disadvantage. There were promising indications of program benefits for groups with repeated exposure to natural disasters.

## Introduction

It is well established that there is an increased risk of mental health problems for both adults and children in the aftermath of a mass trauma event such as a natural disaster [1, 2], arising from direct exposure to the hazard event as well as the associated losses and disruptions in the following months and years. While many children show signs of initial distress in the aftermath of a natural disaster, most children are expected to recover with the support of family, friends and the school community. A significant minority, however, have lingering mental health problems and are in need of additional support to recover and function normally [1, 3]. Pre-disaster experiences of adversity, family circumstances and community levels of disruption are all likely to influence the extent of impact on child health and wellbeing [4]. Even in an event such as the COVID-19 pandemic during which children have been shown to be much less susceptible to the virus than adults, family-level distress and conflicts, overburdened healthcare systems, school closures and social and economic difficulties can still leave children vulnerable. These problems may adversely affect food security, disrupt cognitive and emotional development, impair access to social and medical services and increase the likelihood of exposure to family violence [5, 6].

There has been increasing recognition of the need for appropriate disaster recovery support programs for children and adolescents over the past two decades. These programs can be difficult to operationalise in the upheaval of a post-disaster environment. Given the increasing risk of disasters occurring with more frequency, severity and complexity due to climate change, it is imperative to monitor emerging evidence about which programs are likely to provide the most effective support and which program delivery modes are likely to be feasible and appropriate in post-trauma settings.

The Australian and International Guidelines for the Treatment of Acute Stress Disorder and Posttraumatic Stress Disorder recommend a stepped care approach to post-disaster psychosocial recovery for both children and adults [7]. In the post-disaster context, a stepped care model assumes resilience, but offers recovery support at the community level in the first days and weeks after a disaster, followed by increasingly intensive, targeted, transdiagnostic interventions for those demonstrating ongoing distress and/or who are identified as being at risk of developing a mental health disorder. This approach relies on effective screening and triage practices to ensure those at risk are identified and provided access to the appropriate level of care as early as possible [8].

Level 1 in the stepped care approach, identified as universal care, promotes recovery by offering support, education and advice on self-care strategies such as calming techniques and social connectedness. Psychological First Aid (PFA), based on five essential elements of immediate and mid-term mass trauma intervention identified by Hobfoll et al. [9]—namely safety, connectedness, self and collective efficacy, calm and hope, is an example of a level 1 universal intervention. There are multiple versions and implementation guides for PFA, including directions written specifically for use with children [10, 11].

Level 2 specifies both “selective” and/or “indicated” interventions that are targeted at those exhibiting continued signs of distress or sub-clinical signs of a mental health disorder in the months following the disaster. These early intervention programs usually offer some skills training in adaptive coping aimed at building resilience and reducing the risk of developing a posttraumatic mental health disorder. An example of a level 2 intervention developed by an international panel of trauma experts for use with children, adolescents and adults is Skills for Psychological Recovery (SPR) [12]. Another is Skills for Life Adjustment and Resilience (SOLAR) [13]. Appropriately, trained health care professionals or community support personnel can deliver these interventions at either a primary care or community level.

Level 3 interventions are high-intensity, evidence-based psychological therapies aimed at treating diagnosed posttraumatic mental health disorders and must be delivered by specialist mental health professionals. Interventions with the strongest evidence base are Trauma-Focused Cognitive Behavioural Therapies and can be transdiagnostic in nature or targeting a specific disorder.

While the stepped care approach is widely endorsed by trauma specialists and treatment guidelines internationally, the evidence supporting its effectiveness has been slow to develop, particularly regarding level 1 and 2 and child-focussed interventions. However, recent meta-reviews have identified a range of post-disaster psychological and psychosocial interventions for children and adolescents that demonstrate those receiving interventions fared better than those in control or waitlist groups [14–16]. The strongest evidence available was for eye movement desensitization therapy (EMDR), Exposure and Strict Cognitive Behavioral Therapy (CBT) in level 3 interventions [14]. While pre-post studies present evidence of stronger effects when programs were delivered by qualified professionals, and when delivered to individuals rather than groups, these differences in effect sizes were lower or absent in the controlled studies [15]. A meta-review of level 1, school-based programs delivered post disaster and published between 2000 and 2015 [16] showed that “school-based, universal programmes that are conducted by teachers or local paraprofessionals are effective in reducing PTSD symptoms in children and adolescents” (p. 161). This finding of the effectiveness of school-based programs is also supported by a previous meta-analysis of school-based (level 3) treatment programs targeted at reducing symptoms of PTSD arising from exposure to various forms of trauma including disaster [17]. All the reviews used measures of psychological distress or PTSD as the outcome measure regardless of whether the intervention was universal (level 1), targeted (level 2) or treatment (level 3). Arguably, measures of PTSD symptoms or any other diagnosable mental health disorder are not appropriate for a level 1 intervention given those interventions do not target specific disorders. Even if used as a screening tool, students with sub-clinical symptoms should be directed to a level 2 intervention and those with more severe symptoms to a level 3 intervention. A common recommendation was to conduct further studies with larger samples. Brown and colleagues [15] suggested that the evidence supported a stepped care approach that provides individual treatments for those with high need and a small number of group treatment sessions for those with lesser needs.

The challenges associated with conducting clinical trials and evaluating the effectiveness of interventions in post-disaster environments are well-known, with multiple factors contributing to the complexity, not least of which is the heterogeneity of program models offered and a lack of identified consistent outcomes and goals [18, 19]. Shultz and Forbes (18, p. 8) outline several questions and processes that might be used as a framework to guide evaluation of PFA. At a minimum, they suggest that “The “five essential elements” identified by Hobfoll and colleagues (safety, calming, connectedness, self-efficacy and hope) might be considered the best “standard” available for assessing the coverage of various PFA frameworks.” These elements were developed through expert consensus to guide intervention and prevention efforts following mass trauma events. They were developed in 2007 by extrapolating from related fields of research in the absence of direct evidence at the time. Using them now to review emerging evidence provides the dual benefit of providing a structure for differentiating between available interventions, while also building the evidence for each of the principles.

The goal of this scoping review is to identify any emerging psychosocial interventions and/or new evidence regarding existing disaster recovery programs for children and adolescents that would help inform best practice. The review employs the stepped care model as a structure for differentiating the intervention studies and the presentation of findings.

## Methods

This review was conducted using a scoping review approach informed by Arksey and O’Malley [20]. This approach was consistent with the review’s aim to explore recent trends and developments across a wide range of interventions that were designed and assessed based on an array of theoretical and methodological frameworks.

The final search was conducted on 18 May 2021 across the following databases: Cochrane Library, EMBASE, Family & Society Studies Worldwide, Global Health, Medline, PILOTS (Published International Literature on Traumatic Stress), PsycINFO, Scopus, SocINDEX, and Web of Science and article reference lists. In keeping with the journal focus, the review scope was studies published in the last 3 years.

Inclusion criteria are articles that (a) are peer-reviewed primary research or reviews of primary research, (b) are published in English, (c) are published between 1 January 2018 and 18 May 2021, (d) assess interventions implemented in the aftermath of a natural^1^ disaster, (e) target interventions focusing on child mental health (understanding children as all people under 18 years old).

The review data were categorised according to the stepped care model and the literature on the key elements of interventions for disaster-affected communities. Two team members (KM and LT) developed the evidence table with six test articles. They then independently extracted information from all included articles based on the following categories:

- Study details (reference, organisations involved, name of program/intervention, country/region, type of disaster)
- Level of intervention in the stepped care model
- Elements of interventions (program features, participants and scale, program modules and modality, delivery mode, provider credentials, costs, level of evidence for the program and barriers).
- Alignment of intervention with one or more of the five essential elements of recovery—i.e. safety, calming, connectedness, efficacy and hope.
- Discrepancies in study selection and data extraction were resolved in collaboration with other members of the research team (LG and JN). The final step was to collate, summarize and synthesize the extracted information, based on the following guiding questions:
- When and where were the interventions implemented?
- What type of interventions were delivered?
- How did the interventions align with the five essential elements of disaster recovery?
- How were the interventions delivered?
- Who received the interventions?
- What intervention evaluation study designs were used?
- What were the outcomes and impact of the interventions?

## Results

**Fig. 1:**
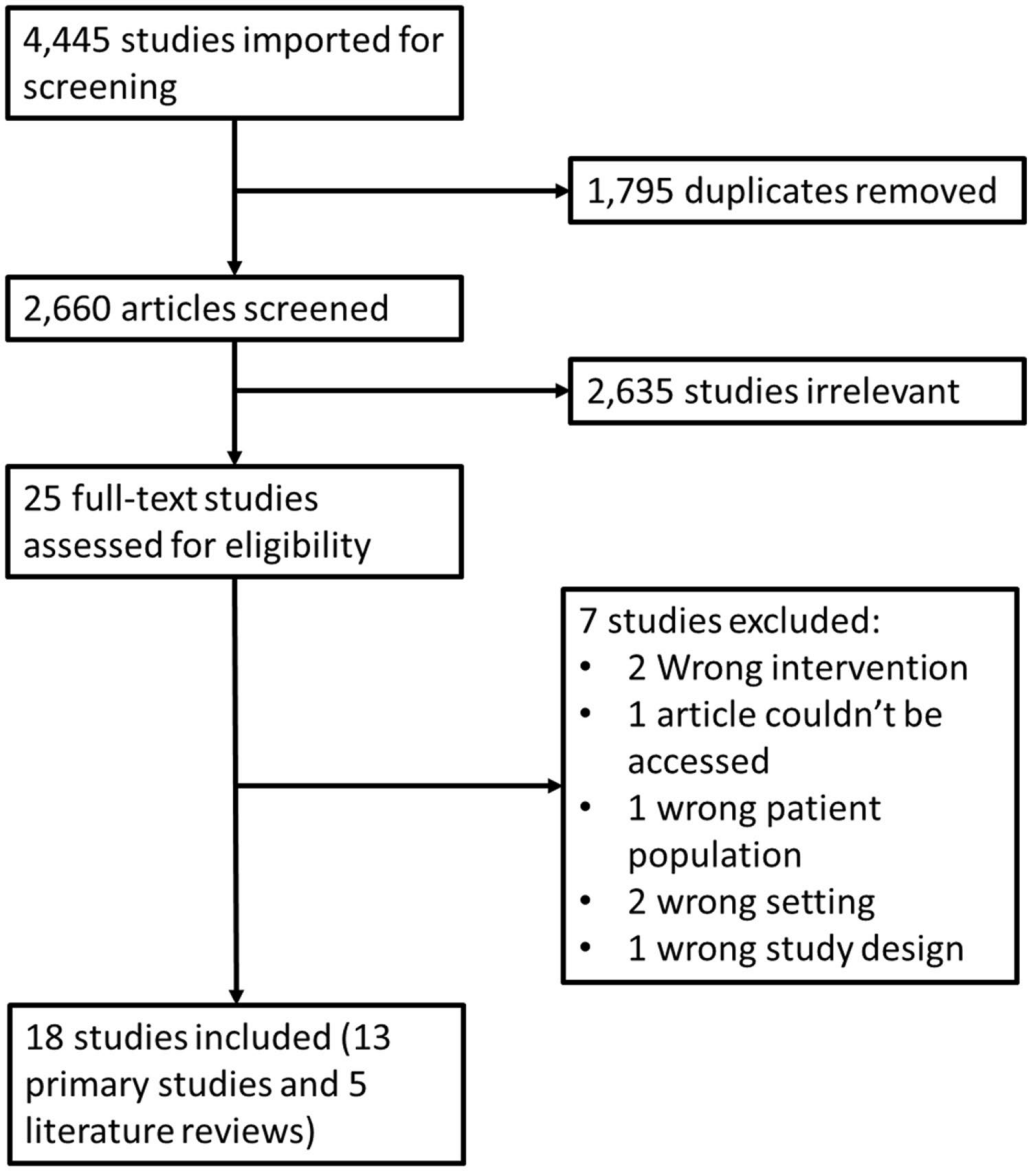
Flow chart for the process of study selection

A total of 18 studies were identified, including 13 primary research articles and 5 literature reviews (see Fig. 1).

The 5 literature reviews aimed to compare the effects of different interventions on PTSD, depression, and anxiety [21, 22•, 23, 24••], and identify the factors that influence the delivery and effectiveness of interventions [22•, 24••]. In terms of the nature of the event, one review [21] focused on different types of natural disasters, while the rest explored interventions delivered in a wider range of potentially traumatic experiences [22•, 23, 24••], including disasters, sexual and physical abuse, war, terrorism and other humanitarian crisis. Additionally, one review [25] focused on identifying interventions for children and families in the context of COVID-19 and comparable outbreaks. No further description of the reviews will be provided in these results because they assessed studies that were either conducted prior to 2018 or were captured in this scoping review. However, the literature reviews will be referred to throughout the discussion to indicate similarities and differences in the findings that have emerged from the more recent primary research studies.

The 13 primary research studies corresponded to 13 different interventions and will now be described in detail in the results below.

### When and Where Were the Interventions Implemented?

The studies identified by this review were carried out in different countries across Asia [26, 27•, 28, 29•, 30, 31], the Americas (four, with two interventions from the USA [32, 33] and two from Canada [34, 35], Europe [36•], Africa [37] and Oceania [38]).

Table 1 summarizes the main characteristics of the interventions identified in this review. The interventions were carried out between 2006 and 2020, although four studies did not report when the post-disaster intervention occurred (and how long after the disaster) or its duration. Four interventions were implemented while the COVID-19 pandemic was happening [30, 32, 34, 35], two interventions were carried out within a few months of a disaster [29•, 37] and three interventions were implemented 1 year after the disaster or shortly after the first anniversary [26, 33, 38].

**Table 1:** Summary of interventions and their main characteristics

Level of intervention	Reference	Intervention	Location	Type of disaster/year	Setting	Time of implementation	Aim	Activities	Duration of intervention	Delivery schedule
**Level 1**	Akiyama et al. [26]	Mastery Approach to Coaching (MAC)	Leyte, Philippines	Typhoon Haiyan (2003)	School/in person	January–February 2015, 14 months after the disaster	Improving self-esteem	Sports (volleyball), intra-school tournaments	2 months	Recommended minimum of 1 h a day, 4 times per week
	Decosimo et al. [37]	Playing to live	40 sites across Liberia	Ebola pandemic (2013–2015)	Community/in person	May–November 2015, approximately 6 months after the Ebola pandemic	Building healthy relationships, trauma coping skills and a safe space for children to express themselves	Art, play, and storytelling to explore what children want for their future	7 months	2–3 times per week
	Hasanudin et al. [28]	Therapeutic group therapy (TKT)	East Java, Indonesia	Kelud Mountain disaster (volcanic eruption) (2014)	School/in person	Information not available	Promoting self-efficacy	Games and group discussions	Information not provided	Information not provided
	Malboeuf-Hurtubise et al. [35]	Online philosophy for children and mindfulness interventions	Eastern Townships region, Quebec, Canada	COVID19 pandemic (2020)	School/online	May–June 2020, during the COVID-19 pandemic	Reducing anxiety, depression, inattention and hyperactivity symptoms	Emotion-based directed drawing, drawing of mandalas	5 weeks	5 weekly sessions of 45 min each
	Malbouef-Hurtubise et al. [34]	Online art therapy	Eastern Townships region, Quebec, Canada	COVID19 pandemic (2020)	School/online	May–June 2020, during the COVID-19 pandemic	Reducing anxiety and inattention symptoms and improving basic psychological needs satisfaction	Philosophy for children, group discussions, mindfulness exercises	5 weeks	5 weekly sessions of 45 min each
	Osofsky et al. [33]	Youth Leadership Program (YLP)	New Orleans, USA	Hurricane Katrina (2005)	School/in person	2006–2008, 2 years after the disaster	Improving student wellbeing	Volunteering, disaster risk reduction, summer program, 1 leadership summit	2 years	Varied across time, groups and schools
**Level 2**	Amin et al. [27•]	Support for Students Exposed to Trauma (SSET) program	Multan, Southern Punjab, Pakistan	Successive floods	School/in person	August 2015–February 2016	Reducing PTSD symptoms, building resilience and social support	Psychoeducation, relaxation techniques, cognitive restructuring, behavioural exposure, social problem-solving	10 weeks	10 weekly sessions of 45 min each
	Ding and Yao [29•]	Model 328 peer education intervention	Hunan and Guangxi provinces, China	COVID-19 (2020)	School/online	February–March 2020, during the COVID-19 pandemic	Improving mental health	Youth-led online live webcasts and aerobic exercise	2 months	Twice a day, 3 times a week
	Yuan [30]	Online mindfulness intervention	China	COVID-19 (2020)	School/online	February 2020, during the COVID-19 pandemic	Improving resilience and emotional intelligence	Mindfulness exercises	Information not provided	15 min a day for 6 months
	Yustiana et al. [31]	Group play therapy	Banten Province, Indonesia	Earthquake and tsunami (2019)	School/in person	Information not available	Reducing PTSD symptoms	Group play therapy	Information not provided	Information not provided
**Level 3**	Lee and Simpson [32]	Three-Step, Single Session Therapy Intervention	United States of America	COVID19 pandemic (2020)	Paediatric emergency department/in person	Information not available	Reducing anxiety symptoms	Psychoeducation, cognitive and behavioural techniques, goal setting	1 session	10 sessions (20–45 min) for young people, 5–6 sessions for parents, completed at their own pace
	Stasiak et al. [38]	BRAVE-ONLINE (cognitive-behavioural therapy program)	Christchurch, New Zealand	Canterbury Earthquakes (February 2011), followed by over 10,000 aftershocks in the following 18 months	School/online	Between 2012 and 2013, 14 and 20 months after the disaster	Anxiety management	Standard CBT anxiety management techniques in two versions: 7–12 and 13–18 years old. Psychoeducation for parents	12 weeks	3 weekly sessions, 60–90 min each
	Trentini et al. [36•]	EMDR Integrative Group Treatment Protocol (EMDR-IGT)	Town of Norcia and surrounding villages in the Umbria region, Italy	2 consecutive earthquakes (2016)	School/in person	Information not available	Reducing PTSD and emotional distress	EMDR sessions	3 weeks	10 sessions (20–45 min) for young people, 5–6 sessions for parents, completed at their own pace

Interventions for child mental health were performed in response to pandemics—mostly COVID-19 [29•, 30, 32, 34, 35, 37], earthquakes [31, 36•, 38], floods [27•], hurricanes [33], tsunamis [31], typhoons [26] and volcanic eruptions [28]. Ten out of thirteen interventions were carried out in school contexts [26, 27•, 28, 29•, 30, 31, 33–35, 36•]. Eight interventions were delivered face to face [26, 27•, 28, 31–33, 36•, 37], while the rest were online [29•, 30, 34, 35, 38]. Almost all the online interventions [29•, 30, 34, 35] were designed in response to the challenges of accessing children during the COVID-19 pandemic in 2019–2020, with one exception that was delivered after an earthquake [38]. This was the only study to report problems caused by frequent technological glitches and high attrition rates.

Most interventions were delivered by clinically trained personnel and mental health professionals [27•, 31–33, 36•, 38], or by researchers with a background in mental health [28, 29•, 34, 35]. Four studies reported that interventions were delivered by or with the support of local teachers [26, 30, 33] or community workers [37], but did not provide details on whether these deliverers had also been affected by the disaster themselves.

### What Type of Interventions Were Implemented?

The interventions identified in this review were based on a diverse set of frameworks, drawing mostly from therapeutic approaches like cognitive-behavioural therapy [27•, 32, 38], EMDR [36•], art therapy [35, 37], yoga therapy, play therapy, child development [37] and group therapy [28, 31], but also from mindfulness [30, 34], philosophy for children [34], health promotion and education [29•, 33], community-based interventions [33], coaching [26] and peer education [29•] (see Appendix 1).

Table 1 shows that the most common aim for interventions was to reduce symptoms of psychological distress (e.g. anxiety, mood, inattention and hyperactivity) and improve coping skills [27•, 31, 32, 34, 35, 36•, 37]. Other interventions aimed to promote resilience and emotional intelligence [27•, 28, 30], social support [27•, 29•], self-expression [37], self-efficacy [28] and self-esteem [26]. Only one intervention explicitly aimed to engage children and youth in disaster recovery activities [33].

Six interventions corresponded to level 1 in the stepped care model [26, 28, 33–35, 37], four interventions were classified as level 2 [27•, 29•, 30, 31] and three interventions corresponded to level 3 [32, 36•, 38]. Most interventions (*N* = 10) were delivered in groups [26, 27•, 28, 29•, 31, 33–35, 36•, 37] and three were delivered to individual children [30, 32, 38]. The activities used to promote mental health varied greatly across interventions. They included sports [26, 29•], psychoeducation [27•, 32, 38], mindfulness, meditation or relaxation techniques [27•, 30, 34], cognitive and behavioural restructuring techniques [27•, 32, 38], art [35, 37], play [31, 37], group therapy techniques [28], philosophy discussions [34], volunteering in the community [33], engaging in disaster recovery [33], and EMDR group sessions [36•]. Most interventions had fixed contents, meaning they were designed to deliver standardised content in a standardised format [26, 27•, 28, 29•, 30, 34, 35, 37, 38], although researchers in one study reported that the frequency of sessions could not be kept the same across sites [26]. In two other studies, researchers reported that the intervention was outlined in broad terms and then tailored to the needs of the community [33] or individual patient being targeted [36•].

### How Did the Interventions Align with the Five Essential Elements of Disaster Recovery?

**Fig. 2:**
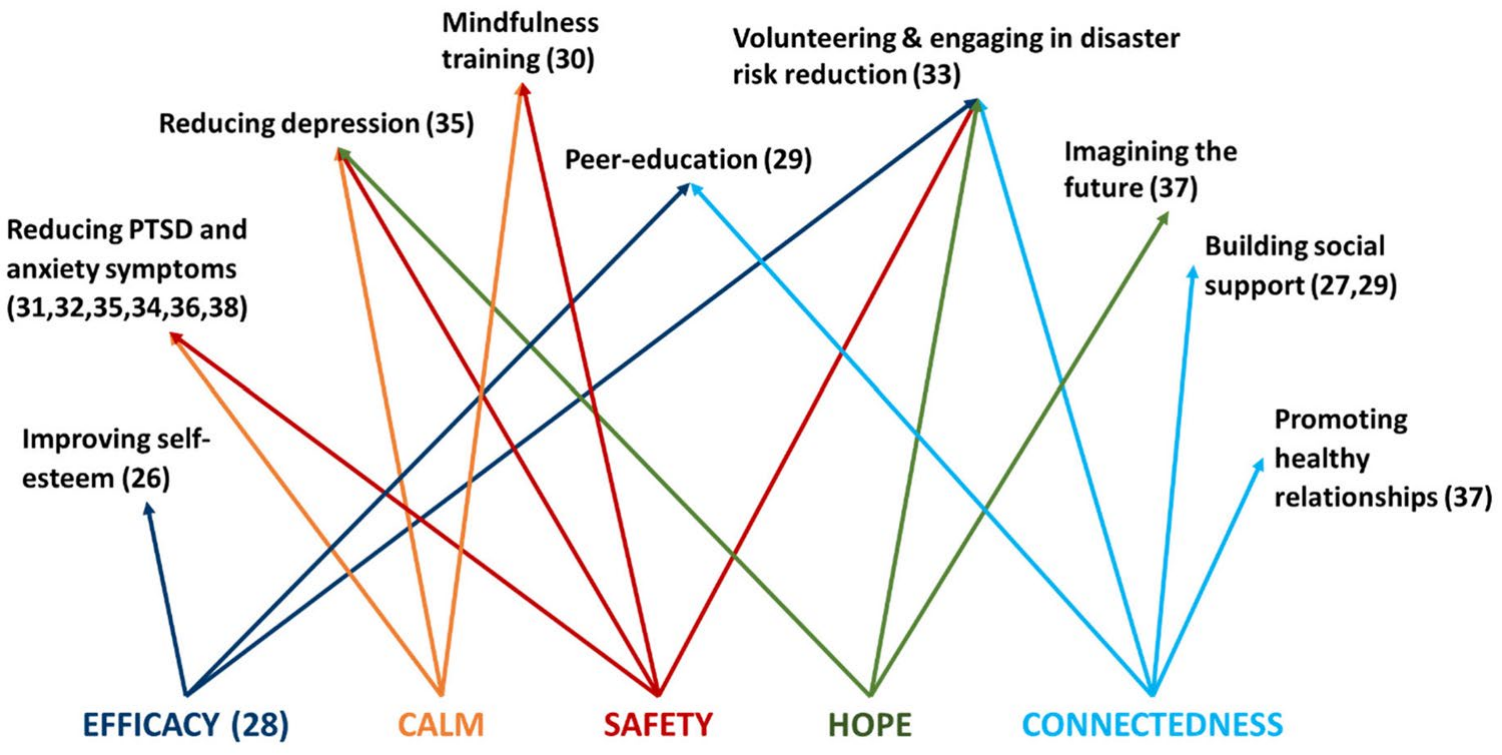
Intervention alignment with the five essential elements of disaster recovery

Figure 2 shows how the different interventions aligned with the five essential elements of disaster recovery (safety, calm, connectedness, efficacy, hope) [9]. Only one intervention [28] explicitly stated an intent to address one of the five elements: self-efficacy. However, for the rest of the interventions, it was possible to link their objectives with different elements.

From this perspective, most interventions served to promote a sense of safety (*N* = 9), followed by calm (*N* = 8), connectedness (*N* = 5), efficacy (*N* = 4) and hope (*N* = 3). All the interventions that addressed connectedness [27•, 29•, 33, 37] and hope [33, 34, 37] were classified as either level 1 or 2 interventions. Some interventions also included additional components such as physical health aspects (sleep and exercise).

### How Were the Interventions Delivered?

The duration of interventions ranged from a single session to 2 years, with the majority lasting about two months (Table 1). The scale of interventions varied from a sub-section of a school [34, 35], to school-wide [26, 33], to different locations within a city [38], region or nation [37] (Appendix 1).

Most interventions involved multiple sessions at regular intervals, that lasted between 45 and 60 min (Table 1). Most interventions had weekly sessions [27•, 33–35, 36•], and three had more than one session per week (between 2 and 4) [26, 29•, 37]. Exceptions included, a single-session intervention [32], a 10-session online self-paced intervention [38] and brief daily mindfulness exercises [30].

### Who Received the Interventions?

All the interventions identified in this review worked directly with children to support their mental health and almost all of them (*N* = 10) were delivered to groups of children [26, 27•, 28, 29•, 31, 33–35, 36•, 37] (Appendix 2). Some targeted children between ages 7 and 13 [27•, 34, 35]; others worked with different ages and stages [31, 33, 37] but did not report how they tailored activities to the different ages, except one intervention [38] which reported two different modes of delivery: for ages 7–12 years and for 13–18 years. Three studies [28, 31, 36•] did not report the age of the children receiving the intervention. No interventions were specially developed for preschool children. Five interventions also offered support and information to parents/caregivers [27•, 32, 37, 38] or teachers [31].

### What Intervention Evaluation Study Designs Were Used?

A range of study designs were used to assess the impact of the interventions (see Table 2). The randomised experimental trials and randomised cluster trials provide the greatest strength of evidence in terms of study design [27•, 29•, 34, 35] but sample sizes were small (ranging from 22 to 141 participants). Quasi-experimental trials were also common [26, 28, 30, 31, 33] (where participants are not randomly assigned to the intervention or the control group). Importantly, only one study [33] carried out a longitudinal analysis of the impacts of an intervention 2 years after the disaster.

**Table 2:** Summary of evaluation design and methods

Level of intervention	Reference	Methodology	Sample size	Outcomes	Outcome measures	Level of exposure measures
**Level 1**	Akiyama et al. [26]	Quasi-experimental trial	293	Self-esteem (self-reported)	Rosenberg’s Self-esteem Scale	None
	Decosimo et al. [37]	Uncontrolled experimental trial	233	Psychological stress symptoms (self-reported)	Self-report locally developed checklist to assess withdrawal, extreme anger, bedwetting, worry/anxiety, poor eating habits, violence and continual sadness	None
	Hasanudin et al. [28]	Quasi-experimental trial	56	Resilience (self-reported)	Information not provided	None
	Malboeuf-Hurtubise et al. [35]	Randomised cluster trial	22	Mental health difficulties (self-reported) Emotional Intelligence (self-reported) Basic psychological need (BPN) satisfaction (self-reported)	Selected items related to anxiety and inattention from Behavior Assessment Scale for Children-3rd edition, self-report scale (BASC III) (Reynolds and Kamphaus, 2004) BPN satisfaction 5-item scale developed by the research team	None
	Malbouef-Hurtubise et al. [34]	Randomised cluster trial	37	Mental health symptoms (self-reported) Mindfulness (self-reported)	Behavior Assessment Scale for Children-3rd edition (BASC III) Reynolds & Kamphaus (2004) Mindful Attention Awareness Scale for Children awlor (Schonert-Reichl, Gadermann & Zumbo, 2014)	None
	Osofsky et al. [33]	Quasi-experimental longitudinal study based on secondary data	212	Self-efficacy (self-reported) Trauma Symptoms (self-reported)	Self-reported Status Questionnaire for Students (specially developed for this study) Locally adapted version of the National Child Traumatic Stress Network Disaster Interview	National Child Traumatic Stress Network Disaster Interview: yes/no questionnaire for students Yes/no questionnaire about additional trauma exposure (other than disaster)
**Level 2**	Amin et al. [27•]	Randomised experimental trial	75	PTSD symptoms (self-reported) Social Support (self-reported) Resilience (self-reported)	Child PTSD symptoms Scale-V (CPSS) (Foa, Johnson, Feeny & Treadwell, 2001) Child and Adolescent Social Support Measure (CASSS; Malecki, Demaray, Elliot & Nolten, 1999) Child and Youth Resilience Measure (CYRM-28) (Ungar & Liebenberg, 2009)	None
	Ding and Yao [29•]	Randomised experimental trial	141	Anxiety (self-reported) Depression (self-reported) Sleep patterns (self-reported)	Self-rating anxiety scale (SAS) (Zung 1971) Self-rating depression scale (SDS) (Zung 1965) Pittsburgh Sleep Quality Index (PSQI) (Buysse et al. 1989)	None
	Yuan [30]	Quasi-experimental trial *(only the control group was randomly assigned)*, latent growth modelling	174	Resilience (self-reported)	Connor–Davidson Resilience Scale (CD-RISC), translated and adapted to the Chinese context by (Yu & Zhang, 2007) Emotional Intelligence Scale (EIS) by Schutte et al (1998, 2001) and translated by Wang (2002)	None
	Yustiana et al. [31]	Quasi-experimental trial	25 students (23 were under 18 years old)	PTSD symptoms	Information not provided	None
**Level 3**	Lee and Simpson [32]	Case study	1	Anxiety and somatic symptoms (assessed through clinical interview)	Child and parents’ report of anxiety and somatic symptoms	None
	Stasiak et al. [38]	Pragmatic open feasibility trial	42	Anxiety (assessed through clinical interviews) Anxiety (self-reported) Depression (self-reported) Health-related quality of life (self-reported) Overall functioning (self-reported) Program satisfaction (parents and children) (self-reported)	Anxiety Disorders Interview Schedule for DSM-IV: Child and Parent versions (ADIS-C/P) Spence Children’s Anxiety Scale (SCAS) (Spence, 1997) Mood & Feelings Questionnaire (MFQ-S) Short version (Angold et al., 1995) Child Health Utility 9D (CHU9D) Children’s Global Assessment of Functioning (CGAS) (Shaffer et al., 1983) 8-item questionnaire used by the program developers (Spence et al., 2006)	None
	Trentini et al. [36•]	Uncontrolled experimental trial, linear mixed-model repeated measures	332	Emotional disorders (self-reported) PTSD (self-reported)	Emotion Thermometers (ET-5) (ET-5; Mitchell et al., 2010; Italian translation) The Children’s Revised Impact of Event Scale (CRIES-13; Perrin et al., 2005)	None

Table 2 also shows that the sample size varied greatly across all the studies, from 1 to 332 children. Small sample size was a common limitation reported by studies [26, 34, 35]. Most studies reported the age and gender of children and aimed to achieve a balance between female and male participants. Only three studies reported on the involvement of children from minority groups [27•, 33, 38]. None of the studies reported involvement of children with disabilities. Only two studies [27•, 38] reported the socioeconomic status (SES) of their participants but did not use that data to examine the effect of SES or to adjust for SES in assessment of intervention impacts.

Table 2 also shows the interventions according to their expected outcomes and measures, demonstrating the wide range of standardised measures used to evaluate outcomes related to different aspects of children’s mental health, behaviour and wellbeing. Only one study [33] incorporated a measure of children’s level of disaster exposure before receiving the intervention.

### What Were the Outcomes and Impact of the Interventions?

Considering the study limitations in strength of evidence as described in the previous section, the studies considered in this review suggested an overall positive impact of the different interventions in terms of reducing PTSD symptoms, depression, anxiety, sleep problems and promoting resilience, perceived social support and self-efficacy [26, 27•, 28, 29•, 31–35, 36•, 37].

However, findings were often mixed in terms of which aspects of the interventions were most effective and which sub-groups benefitted the most. One study reported that the intervention using group play therapy helped decrease PTSD symptoms including intrusions, avoidance, negative alterations in cognitions and mood, and alterations in arousal and reactivity [31], and another study [36•] found that EMDR group interventions were more effective in females and older children. While most level 2 and 3 studies included a measure of PTSD symptoms, almost all the PTSD and other outcomes were determined using self-report measures. Only two level 3 studies [32, 38] determined anxiety symptoms through diagnosis from a specialized clinician.

In relation to interventions that also had a component to support teachers [31] or parents/caregivers [27•, 32, 38], only one study [31] assessed the impact of the intervention on adults’ mental health. However, this evaluation measured the impact of the intervention on teachers and students together, so it is not possible to make any conclusions about the intervention’s effects on adults.

In addition to limitations already noted in relation to sample size and lack of diversity, study authors reported participant attrition [37], lack of follow up over time [34, 35], variations in the implementation of interventions across different study sites [26], and not assessing other potential sources of support that may have influenced the outcomes of an intervention [30]. Several studies reported not using randomised control groups [33–35, 36•, 37, 38] because of ethical concerns. Limitations of instruments used for measuring intervention effects included uncertainty about reliability of translated questionnaires [26, 28], challenges assessing complex concepts with multiple dimensions like resilience [33] and bias in clinical assessments [38]. Details on cost-effectiveness, inclusiveness, risk management strategies (e.g. mitigating risk of re-traumatisation) and implementation processes were also commonly missing from evaluation reports (see Appendix 3).

## Discussion

This scoping review aimed to identify recent intervention and evaluation trials of post-disaster psychosocial programs for children and adolescents after disasters. The findings revealed studies conducted across five continents, following a wide range of types of natural disasters including major weather events, floods, volcano, pandemic and tsunami. They were delivered to different age groups, with schools being the most common setting for delivery, building on previous reviews of school-based programs [16, 17]. Unfortunately, it appears that the unique mental health needs of children in their preschool years continue to be overlooked [39].

Presenting the review findings within a stepped care framework in which universal interventions were allocated to level 1, targeted interventions to level 2 and treatment interventions to level 3, provided a useful means of differentiating the evidence. More of the recent studies of mental health interventions for children after disasters corresponded to level 1 interventions (*N* = 6) with slightly less defined as level 2 (*N* = 4) or level 3 (*N* = 3). This may manifest a shift towards mental health promotion through community-based interventions, consistent with evidence showing that, after disasters, most children will show signs of resilience, and a few will develop more severe symptoms that require more specialized treatment [1, 3, 15]. While the evidence is building across each level of the stepped care approach, the strength of evidence is still limited due to considerable heterogeneity in intervention strategies, evaluation study design, timeframes and measures used [15]. Study limitations also included challenges achieving adequate sample sizes, participant attrition, lack of diversity, technological problems in online delivery, measurement challenges and lack of differentiation based on socioeconomic status. A meta-analysis identified by this review [24••] concluded that more research is needed on the influence of socioeconomic factors on the effectiveness of mental health interventions for children.

All the interventions were assessed in terms of their outcomes and impacts, and most included some sort of control or comparison group. The overall findings show positive program impacts on the mental health and wellbeing of the children and adolescent participants, consistent with previous reviews [14, 15, 17]. The literature reviews that aimed to compare the impact of level 3 interventions found that CBT was the most beneficial intervention for children [21, 22•, 23], followed by EMDR [22•, 23]. In terms of the factors influencing the impact of interventions, in their meta-analysis, Pfefferbaum and colleagues [24••] investigated a range of factors that might potentially influence treatment effectiveness and found that interventions that had significant effects on depression tended to be carried out in high-income countries and had more than eight sessions and interventions that were non-trauma focused.. Only non-trauma-focused interventions had a significant effect on anxiety symptoms. They noted however that the reliability of these findings was undermined to some extent by the heterogeneity of the studies and a lack of specific information provided about the interventions used.

The evidence on the negative impact of disasters on mental health shows that these effects can be long-lasting [1, 3]. More longitudinal studies are needed to assess the impact of interventions in the mid to long term. Intervention trials conducted with children and adolescents who have experienced multiple disasters are also increasingly important as the onset of climate change increases the likelihood of exposure to more frequent, more severe and more complex disaster scenarios [40]. In this review, most studies involved population groups exposed to a single major disaster event but importantly two of the studies were conducted with children who experienced repeat exposures to the same type of hazard [36•, 38] and one intervention involved students who experienced two different types of disasters—earthquake and tsunami [31], with positive outcomes. It is not clear in the Amin et al. report [27•] if the children and schools included in the study had repeated exposure to the floods or not. Lewey et al. [22•] in their review of EMDR and TF-CBT trials for children and adolescents found no significant differences in the effect sizes of studies for those with exposure to either single or mixed trauma type (chronic or repeated events).

The COVID-19 pandemic is reshaping mental health interventions for children after disasters, with new advances in use of digital technology to teach children and developing telehealth interventions. This was reflected in this review which identified four interventions conducted online during the COVID-19 pandemic. An additional equity consideration for these interventions is the level of access that families have to digital devices and internet connection and provides an example of how local considerations can be important in shaping psychosocial interventions and evaluation of impacts. The importance of stakeholder input into disaster preparedness and recovery initiatives is enshrined in the Sendai Framework for Disaster Risk Reduction [41]. While standardised programs may be more easily replicated across large geographic areas with multiple locations, they can have the disadvantage of not adapting to local and individual resources and needs. Community involvement in intervention planning was a feature of one level 1 study [33] but most interventions were led by organizations from outside the community and the vast majority of the interventions had standardised content rather than tailoring them to different locations or individual children. One study demonstrated capacity to localise to a certain extent by engaging with local community to adapt the intervention to local languages in a level 2 intervention [27•] and another adjusted the treatment protocol to patients’ symptoms in a level 3 intervention [36•]. Only one of the interventions in this review explicitly engaged children and youth in disaster recovery activities [33]. Involvement in disaster recovery planning and activities can be beneficial for young people [3] and this could have a positive influence on self-efficacy, one of the five essential elements of intervention following a mass trauma intervention [9]. Self-efficacy was explicitly addressed by only one of the interventions [28] and none of the other 5 essential elements were named by any of the interventions. However, we propose that each intervention’s aims did align with at least one of the elements—calm, safety, connectedness, efficacy and hope. The most common aim was reducing PTSD, anxiety and depression symptoms, arguably a means of promoting a sense of calm and safety. Conversely, promoting hope and connectedness were the two elements that were addressed by the smallest number of interventions. All of the interventions addressing hope were level 1 [33, 35, 37] and those addressing connectedness were level 2 [27•, 29•] interventions. It may be helpful for future interventions to consider these elements in planning both intervention and evaluation components.

## Conclusions

This scoping review contributes to the growing understanding of the contribution of psychosocial programs to child and adolescent recovery following exposure to natural disasters. Most importantly, it shows that positive impacts are being achieved across a range of programs, delivery modes and settings. Further studies are needed to confirm the findings because there are still a number of limitations to the evidence, not surprisingly given the complexity of post-trauma mental health needs and the disrupted context of post-disaster environments. Structuring the evidence according to a stepped care model provides a useful means of aligning the available evidence with recommended approaches. Similarly, Hobfoll et al.’s [9] nominated five essential elements for intervention following mass trauma, provide a guide for both intervention aims and assessment that is consistent with programs currently being delivered, if not explicitly stated. The most common focus across the interventions, and thus the developing evidence, was promotion of a sense of calm and safety. Unfortunately, current gaps in the evidence relate to potentially the most vulnerable of groups—preschool children, culturally and linguistically diverse groups, children and adolescents with disabilities, and socioeconomic disadvantage. This highlights future research priorities, as well as the need to build further understanding of programs that are feasible and effective in complex, multi-exposure disaster settings.

## Appendix

## Appendix 1 Interventions according to whether developers and deliverers belonged to the disaster-affected community, scale of the intervention, framework and fixed vs tailored contents

**Table:** 

**Level of intervention**	**Reference**	**Where was the intervention designed?**	**Who delivered the intervention? Were program deliverers local or external to the community?**	**Scale of the intervention**	**Framework**	**Fixed vs tailored contents**
**Level 1**	Akiyama et al. [26]	Outside the country	Local teachers	School-wide (across 1 school)	The Mastery Approach to Coaching (MAC), based on goal orientation theory	Fixed
	Decosimo et al. [37]	Same country where the intervention was implemented	Local psychosocial workers and community members	Nation-wide, 40 sites	Art therapy, yoga therapy, play therapy, child development	Fixed
	Hasanudin et al. [28]	Outside the country	Researchers from the same country, but it was not mentioned if they belonged to the community	Information not provided	Therapeutic Group Therapy	Fixed
	Malboeuf-Hurtubise et al. [35]	Same country where the intervention was implemented	Undergraduate psychology students under the supervision of a clinician, it was not mentioned if they belonged to the community	1 classroom in an elementary school	Art therapy and mindfulness	Fixed
	Malbouef-Hurtubise et al. [34]	Same country where the intervention was implemented	Undergraduate psychology students under the supervision of a clinician, it was not mentioned if they belonged to the community	1 classroom in an elementary school	Philosophy for children (P4C) and mindfulness-based interventions (MBIs)	Fixed
	Osofsky et al. [33]	Same community where the intervention was implemented	Local teachers and mental health professionals	School-wide	Community-based and mental health approaches to stress reduction and self-awareness	Tailored to the community
**Level 2**	Amin et al. [27•]	Outside the country	External clinicians with local non-clinical staff	Regional (across 5 elementary public schools in three rural union councils)	Cognitive-Behavioural Intervention for Trauma in Schools	Fixed, but tailored to the local languages
	Ding and Yao [29•]	Same country where the intervention was implemented	Researchers from the same country, but it was not mentioned if they belonged to the community	Across 2 regions in China	Health education, evidence on the effects of exercise on physical and mental health	Fixed
	Yuan [30]	Information not provided	Local teachers	Information not provided	Mindfulness	Fixed
	Yustiana et al. [31]	Information not provided	One researcher from the same country where the intervention was delivered	Information not provided	Group play therapy	Information not provided
**Level 3**	Lee and Simpson [32]	Same country where the intervention was implemented	Clinicians from the Paediatric Emergency Department where the intervention was delivered	One Paediatric Emergency Department	Cognitive-behavioural therapy	Fixed
	Stasiak et al. [38]	Outside the country	The online intervention was implemented with minimal involvement from clinical and occupational therapists	City-wide	Cognitive-behavioural therapy	Fixed
	Trentini et al. [36•]	Outside the country	EMDR therapists working in pairs, who were from the same country or region where the intervention was implemented	Regional	EMDR-IGTP, based on the Standard EMDR Protocol with elements from group and art therapy	Fixed, but the protocol was adjusted to each patient’s symptoms, stage of development and response to treatment

## Appendix 2 Recipients of the interventions identified in this review

**Table:** 

**Level of intervention**	**Reference**	**Age of children who received the intervention**	**Was the intervention delivered individually or in groups?**	**Did children receive the intervention directly, or indirectly through training of teachers or caregivers?**	**Did the intervention include a component to support adults?**
**Level 1**	Akiyama et al. [26]	10th grade students, mean age 16.6 years old	In groups	Directly	No
	Decosimo et al. [37]	4–18 years old	In groups	Directly	No
	Hasanudin et al. [28]	Information not provided	In groups	Directly	No
	Malboeuf-Hurtubise et al. [35]	4th to 5th grade, mean age 11.3 years old	In groups	Directly	No
	Malbouef-Hurtubise et al. [34]	Elementary school students, mean age 8–18 years old	In groups	Directly	No
	Osofsky et al. [33]	9 to 18 years old	In groups	Directly	No
**Level 2**	Amin et al. [27•]	7–13 years old, mean age of 11.43 years	In groups	Directly	Yes, support for parents
	Ding and Yao [29•]	12–18 years old	In groups	Directly	No
	Yuan [30]	12 to 14 years old	Individually	Directly	No
	Yustiana et al. [31]	Under 17 years old	In groups	Directly	Yes, support for teachers
**Level 3**	Lee and Simpson [32]	10 years old	Individually	Directly	Yes, support for parents
	Stasiak et al. [38]	Children aged 7–12 and adolescents aged 13–18 years old	Individually	Directly	Yes, support for parents
	Trentini et al. [36•]	Information not provided	In groups	Directly	No

## Appendix 3 Studies according to whether they reported on the cost-effectiveness, accessibility and inclusion, risk management strategies, implementation and barriers of the intervention

**Table:** 

**Level of intervention**	**Reference**	**Cost-effectiveness**	**Accessibility & inclusion**	**Risk management strategies**	**Implementation**	**Barriers**
**Level 1**	Akiyama, Gregorio, & Kobayashi, J. [26]	No	No	No	Yes, teachers kept a record of how many sessions were carried out at each school site	No
	Decosimo et al. [37]	No	No	No	No	No
	Hasanudin, Arief, Kurnia & Kusumaningrum [28]	No	No	No	No	No
	Malboeuf-Hurtubise et al. [35]	No	No	No	Yes, program fidelity was assessed through clinical supervision	No
	Malbouef-Hurtubise et al. [34]	No	No	No	Yes, program fidelity was assessed through clinical supervision	No
	Osofsky et al. [33]	No	Yes, the program was developed to be inclusive for children who had dropped out of school and/or were not used to leadership roles	No	No	No
**Level 2**	Amin et al. [27•]	No	Yes, based on feedback form the community, the intervention was adapted to Urdu, Punjabi and Siraiki	Yes, based on feedback from the community, the intervention was adapted to allow regular parental contact with deliverers	Yes, fidelity to the program was evaluated through clinical supervision, live observation and surveying co-facilitators	No
	Ding & Yao [29•]	No	No	No	No	No
	Yuan [30]	No	No	No	No	No
	Yustiana, Rusmana & Suryana [31]	No	No	No	No	No
**Level 3**	Lee & Simpson [32]	No	No	No	No	No
	Stasiak, Merry, Frampton & Moor [38]	No	No, two participants had to be excluded because they had a disability	Yes, after an initial screening, potential participants showing moderate to severe levels of depression or anxiety were referred to an appropriate treatment provided by local services	Yes, researchers assessed the number of sessions completed by children and parents and asked for their feedback	Yes
	Trentini [36•]	No	No	No	No	No

## Footnotes

1 We acknowledge the term “natural disasters” is contested because of the human/social influences on these events but use it here as a useful distinction from disasters arising from acts of violence such as terrorism and war.

## References

[34] Malboeuf-Hurtubise C, Léger-Goodes T, Mageau G, Joussemet M, Herba C, Chadi N (2021). Philosophy for children and mindfulness during COVID-19: results from a randomized cluster trial and impact on mental health in elementary school students. Prog Neuropsychopharmacol Biol Psychiatry [Internet].

[35] Malboeuf-Hurtubise C, Leger-Goodes T, Mageau G, Taylor G, Herba C, Chadi N (2021). Online art therapy in elementary schools during COVID-19: results from a randomized cluster pilot and feasibility study and impact on mental health. Child Adolesc Psychiatry Ment Health [Internet].

[36] Trentini C, Lauriola M, Giuliani A, Maslovaric G, Tambelli R, Fernandez I (2018). Dealing with the aftermath of mass disasters: a field study on the application of EMDR integrative group treatment protocol with child survivors of the 2016 Italy earthquakes. Front Psychol [Internet].

[37] Decosimo C, Hanson J, Quinn M, Badu P, Smith C (2019). Playing to live: outcome evaluation of a community-based psychosocial expressive arts program for children during the Liberian Ebola epidemic. Glob Ment Heal [Internet].

[38] Stasiak K, Merry S, Frampton C, Moor S (2018). Delivering solid treatments on shaky ground: feasibility study of an online therapy for child anxiety in the aftermath of a natural disaster. Psychother Res [Internet].

[39] Gibbs L, Snowdon E, Block K, Gallagher HC, MacDougall C, Ireton G, et al. Where do we start? A proposed post disaster intervention framework for children and young people. Pastor Care Educ. 2014;32(1):68–87. 10.1080/02643944.2014.881908

[40] IPCC. Global Warming of 1.5 °C. An IPCC special report on the impacts of global warming of 1.5 °C above pre-industrial levels and related global greenhouse gas emission pathways, in the context of strengthening the global response to the threat of climate change. In press; 2018.

[41] United Nations. Sendai framework for disaster risk reduction 2015–2030 [Internet]. Sendai, Japan; 2015. Available from: https://www.preventionweb.net/files/43291_sendaiframeworkfordrren.pdf

[1] Bonanno G, Brewin C, Kaniasty K, La Greca A (2010). Weighing the costs of disaster: consequences, risks, and resilience in individuals, families, and communities. Psychol Sci Public Interes.

[2] Beaglehole B, Mulder R, Frampton C, Boden J, Newton-Howes G, Bell C. Psychological distress and psychiatric disorder after natural disasters: systematic review and meta-analysis. 213: . Br J Psychiatry. 2018;213:716–722. 10.1192/bjp.2018.210 30301477

[3] Peek L (2008). Children and disasters: understanding vulnerability, developing capacities, and promoting resilience - an introduction. Child Youth Environ.

[4] Norris F, Friedman M, Watson P. 60,000 disaster victims speak: part II. Summary and implications of the disaster mental health research. Psychiatry. 2002;65(3):240–60. 10.1521/psyc.65.3.240.20169 12405080

[5] Ramos G, Scarpetta S. Combatting COVID-19’s effect on children. Tackling Coronavirus (COVID-19): Contributing to a Global Effort. 2020.

[6] Fore HH (2020). A wake-up call: COVID-19 and its impact on children’s health and wellbeing. Lancet.

[7] Phoenix Australia - centre for postraumatic mental health. Australian guidelines for the prevention and treatment of acute stress disorder, posttraumatic stress disorder and complex posttraumatic stress disorder. Melbourne. 2020.

[8] Cohen GH, Tamrakar S, Lowe S, Sampson L, Ettman C, Linas B (2017). Comparison of simulated treatment and cost-effectiveness of a stepped care case-finding intervention vs usual care for posttraumatic stress disorder after a natural disaster. JAMA Psychiat.

[9] Hobfoll S, Watson P, Bell C, Bryant R, Brymer M, Friedman M (2007). Five essential elements of immediate and mid-term mass trauma intervention: empirical evidence. Psychiatry.

[10] Brymer MJ, Taylor M, Escudero P, Jacobs A, Kronenberg M, Macy R, et al. Psychological first aid: field operations guide 2nd edition [Internet]. Los Angeles, CA; 2012. Available from: http://www.nctsn.org/content/psychological-first-aid-schoolspfa

[11] Zoellner, LA, Graham, B, Bedard-Gilligan, MA. Trauma- and stressor-related disorders. In Psychopathology: Foundations for a contemporary understanding, 4^th^ Edition; Routledge; New York; 2016;162–81.

[12] Berkowitz S, Bryant R, Brymer M, Hamblen J, Jacobs A, Layne C, et al. National center for PTSD and national child traumatic stress Network, Skills for Psychological Recovery: Field Operations Guide. 2010.

[13] O’Donnell ML, Lau W, Fredrickson J, Gibson K, Bryant RA, Bisson J, et al. An open label pilot study of a brief psychosocial intervention for disaster and trauma survivors [Internet]. Vol. 11, Frontiers in Psychiatry. 2020. p. 483. 10.3389/fpsyt.2020.00483 10.3389/fpsyt.2020.00483 PMC7332836 32670099

[14] Newman E, Pfefferbaum B, Kirlic N, Tett R, Nelson S, Liles B (2014). Meta-analytic review of psychological interventions for children survivors of natural and man-made disasters. Curr Psychiatry Rep.

[15] Brown RC, Witt A, Fegert JM, Keller F, Rassenhofer M, Plener PL, et al. Psychosocial interventions for children and adolescents after man-made and natural disasters: a meta-analysis and systematic review. Psychological Medicine 47: Psychol Med [Internet]. 2017;47(11):1893–905. 10.1017/S0033291717000496 10.1017/S0033291717000496 28397633

[16] Fu C, Underwood C (2015). Meta-review of school-based disaster interventions for child and adolescent survivors. J Child Adolesc Ment Heal.

[17] Rolfsnes E, Idsoe T (2011). School-based intervention programs for PTSD symptoms: a review and meta-analysis. J Trauma Stress.

[18] Shultz J, Forbes D (2014). Psychological first aid. Disaster Heal.

[19] Wang L, Norman I, Xiao T, Li Y, Leamy M (2021). Psychological first aid training: a scoping review of its application, outcomes and implementation. Int J Environ Res Public Health.

[20] Arksey H, O’Malley L (2005). Scoping studies: towards a methodological framework. Int J Soc Res Methodol.

[21] Galvan MS, Lueke AE, Mansfield L, Smith CA. A systematic research review: how to best treat post-traumatic stress disorder in children post-natural disaster. J Hum Behav Soc Environ [Internet]. 2020; 10.1080/10911359.2020.1804513

[22] Lewey JH, Smith CL, Burcham B, Saunders NL, Elfallal D, O’Toole SK (2018). Comparing the effectiveness of EMDR and TF-CBT for children and adolescents: a meta-analysis. J Child Adolesc Trauma [Internet].

[23] Mavranezouli I, Megnin-Viggars O, Daly C, Dias S, Stockton S, Meiser-Stedman R (2020). Psychological and psychosocial treatments for children and young people with post-traumatic stress disorder: a network meta-analysis. J Child Psychol Psychiatry.

[24] Pfefferbaum B, Nitiema P, Newman B (2019). A meta-analysis of intervention effects on depression and/or anxiety in youth exposed to political violence or natural disasters. Child Youth Care Forum [Internet].

[25] Boldt K, Coenen M, Movsisyan A, Voss S, Rehfuess E, Kunzler AM (2021). Interventions to ameliorate the psychosocial effects of the COVID-19 pandemic on children - a systematic review. Int J Environ Res Public Health [Internet].

[26] Akiyama T, Gregorio ER, Kobayashi J. Youth sports activity and young people’s well-being after a disaster: a trial with the Mastery Approach to Coaching (MAC) in the Philippines. BMC Res Notes. 2018;11(747):(22 October 2018). 10.1186/s13104-018-3860-1 PMC6196409 30348220

[27] Amin R, Nadeem E, Iqbal K, Asadullah MA, Hussain B (2020). Support for students exposed to trauma (SSET) program: an approach for building resilience and social support among flood-impacted children. School Ment Health [Internet].

[28] Hasanudin H, Arief YS, Kurnia ID, Kusumaningrum T (2020). Therapeutic group can increase resilience of school-age children after the Kelud mountain disaster. EurAsian J Biosci [Internet].

[29] Ding X, Yao J (2020). Peer education intervention on adolescents’ anxiety, depression, and sleep disorder during the COVID-19 Pandemic. Psychiatr Danub [Internet].

[30] Yuan Y. Mindfulness training on the resilience of adolescents under the COVID-19 epidemic: a latent growth curve analysis. Pers Individ Dif. 2021;172:110560. 10.1016/j.paid.2020.110560 PMC7831962 33518868

[31] Yustiana Y, Rusmana N, Suryana D (2020). Group play therapy for the treatment of post-traumatic stress disorder in child victim of Tsunami in Banten Province. Elem Educ Online [Internet].

[32] Lee D, Simpson S. A three-step, single session therapy intervention for COVID-related anxiety in a pediatric emergency department. Cureus. 2020;12(12):e12371. 10.7759/cureus.12371 PMC7842244 33527052

[33] •• Osofsky H, Osofsky J, Hansel T, Lawrason B, Speier A. Building resilience after disasters through the youth leadership program: the importance of community and academic partnerships on youth outcomes. Prog Community Heal Partnerships Res Educ Action [Internet]. 2018;12(Special Issue):11–21. 10.1353/cpr.2018.0017. **This quasi-experimental study, incorporating a longitudinal analysis, engaged stakeholders in shaping the intervention and included youth in disaster recovery activities.** 10.1353/cpr.2018.0017 29755045

